# The Protective Role of Adiponectin for Lipoproteins in End-Stage Renal Disease Patients: Relationship with Diabetes and Body Mass Index

**DOI:** 10.1155/2019/3021785

**Published:** 2019-02-18

**Authors:** Susana Coimbra, Flávio Reis, Sara Nunes, Sofia Viana, Maria João Valente, Susana Rocha, Cristina Catarino, Petronila Rocha-Pereira, Elsa Bronze-da-Rocha, Maria Sameiro-Faria, José Gerardo Oliveira, José Madureira, João Carlos Fernandes, Vasco Miranda, Luís Belo, Alice Santos-Silva

**Affiliations:** ^1^UCIBIO/REQUIMTE, Porto, Portugal; ^2^CESPU, Institute of Research and Advanced Training in Health Sciences and Technologies (IINFACTS), Gandra-Paredes, Portugal; ^3^Institute of Pharmacology & Experimental Therapeutics, Coimbra Institute for Clinical and Biomedical Research (iCBR), Faculty of Medicine, CNC.IBILI Consortium & CIBB Consortium, University of Coimbra, Coimbra, Portugal; ^4^Polytechnic Institute of Coimbra, ESTESC-Coimbra Health School, Pharmacy, Coimbra, Portugal; ^5^UCIBIO/REQUIMTE, Laboratory of Biochemistry, Department of Biological Sciences, Faculty of Pharmacy, University of Porto, Porto, Portugal; ^6^Health Science Research Centre, University of Beira Interior, Covilhã, Portugal; ^7^Hemodialysis Clinic Hospital Agostinho Ribeiro, Felgueiras, Portugal; ^8^Hemodialysis Clinic of Porto (CHP), Porto, Portugal; ^9^Center for Health Technology and Services Research (CINTESIS), Faculty of Medicine, University of Porto, Porto, Portugal; ^10^NefroServe, Hemodialysis Clinic of Barcelos, Barcelos, Portugal; ^11^NefroServe Hemodialysis Clinic of Viana do Castelo, Viana do Castelo, Portugal; ^12^Hemodialysis Clinic of Gondomar, Gondomar, Portugal

## Abstract

Cardiovascular disease (CVD) events are the main causes of death in end-stage renal disease (ESRD) patients on dialysis. The number and severity of CVD events remain inappropriate and difficult to explain by considering only the classic CVD risk factors. Our aim was to clarify the changes and the relationship of lipoprotein subfractions with other CVD risk factors, namely, body mass index (BMI) and adipokines, inflammation and low-density lipoprotein (LDL) oxidation, and the burden of the most prevalent comorbidities, diabetes mellitus (DM) and hypertension (HT). We studied 194 ESRD patients on dialysis and 22 controls; lipid profile, including lipoprotein subpopulations and oxidized LDL (oxLDL), C-reactive protein (CRP), adiponectin, leptin, and paraoxonase 1 activity were evaluated. Compared to controls, patients presented significantly lower levels of cholesterol, high-density lipoprotein cholesterol (HDLc), LDLc, oxLDL, and intermediate and small HDL and higher triglycerides, CRP, adiponectin, large HDL, very-low-density lipoprotein (VLDL), and intermediate-density lipoprotein- (IDL) B. Adiponectin levels correlated positively with large HDL and negatively with intermediate and small HDL, oxLDL/LDLc, and BMI; patients with DM (*n* = 17) and with DM+HT (*n* = 70), as compared to patients without DM or HT (*n* = 69) or only with HT (*n* = 38), presented significantly higher oxLDL, oxLDL/LDLc, and leptin and lower adiponectin. Obese patients (*n* = 45), as compared to normoponderal patients (*n* = 81), showed lower HDLc, adiponectin, and large HDL and significantly higher leptin, VLDL, and intermediate and small HDL. In ESRD, the higher adiponectin seems to favor atheroprotective HDL modifications and protect LDL particles from oxidative atherogenic changes. However, in diabetic and obese patients, adiponectin presents the lowest values, oxLDL/LDLc present the highest ones, and the HDL profile is the more atherogenic. Our data suggest that the coexistence of DM and adiposity in ESRD patients on dialysis contributes to a higher CVD risk, as showed by their lipid and adipokine profiles.

## 1. Introduction

End-stage renal disease (ESRD) patients present high morbidity and mortality rates, and the main causes of death are cardiovascular disease- (CVD) related events [1, 2]. Diabetes mellitus (DM) and hypertension (HT) are the most common causes for renal failure and are known as risk factors for CVD events.

ESRD patients on hemodialysis (HD) frequently present normal or decreased values of total cholesterol (TC) and low-density lipoprotein cholesterol (LDLc), decreased high-density lipoprotein cholesterol (HDLc), and increased triglyceride (TG) values [3]. A high prevalence of some traditional CV risk factors is common, though the number and severity of CVD events remain inappropriate and hard to explain when considering only the classic CVD risk factors. A study by our group showed that TG and C-reactive protein (CRP) levels, as well as the type of vascular access, were independent risk factors for all-cause mortality in ESRD patients on HD [4]. It appears that other factors may underlie the inappropriately high CVD events in ESRD patients, namely, oxidative stress/inflammatory-related changes, adipocyte-related cytokines, and altered functionality in lipoproteins.

LDL and HDL are a heterogeneous population of particles with different atherogenic or atheroprotective properties. It has been hypothesized that lipoprotein's quality (size, composition, and functionality) may be more important than their total circulating levels, as a CVD risk factor. In fact, smaller and denser LDL subpopulations are more atherogenic than larger LDL subpopulations [5, 6]. In addition, a reduction in HDL size has been also associated with less functionality [7–9]; however, this issue is not consensual [6]. A study of incident diabetic patients showed lower percentages of large HDL particles, higher percentages of small LDL subfractions, and a reduction in LDL size [10].

A large number of studies evaluated lipid risk profile in ESRD; however, the studies concerning the lipoprotein subfractions, evaluated by high-resolution electrophoresis on polyacrylamide gel tubes, are sparse, with some controversial results and involving a small number of patients [11–13]. These studies reported that ESRD patients, as compared to controls, present increased large HDL particles and decreased small HDL particles [11–13], while data on LDL subfractions is controversial. Moreover, the interplay between lipoprotein quality, oxidative stress, inflammation, and adipokines was not addressed in these studies.

Oxidation of LDL (oxLDL) is a triggering factor for initiation and progression of atherosclerosis. The oxLDL/LDL ratio, used to measure the value of LDL oxidation within LDL particles, seems to be raised in ESRD patients, showing a lower antioxidant protection for LDL particles [14]. ESRD patients on dialysis also present reduced paraoxonase 1 (PON1) activity that has been associated with inflammation, longer time on dialysis, and high recombinant human erythropoietin doses [14].

Obesity is a CVD risk factor and a risk factor for development of chronic kidney disease (CKD) and diabetes. Adiponectin, mainly synthesized by adipocytes, with anti-inflammatory, insulin-sensitizing, and antiatherogenic properties, is increased in ESRD patients and seems to present a negative correlation with body mass index (BMI) in these patients [15, 16]. Hyperleptinemia has been associated with inflammation, insulin resistance, and protein energy wasting and with progression of CKD [17].

Considering the few data on lipoprotein subpopulations in ESRD and, particularly, on their potential implications on morbidity and mortality, the aim of this work was to further clarify the changes in lipoprotein profiles and, particularly, the relationship with other CVD risk factors, namely, BMI and adipokine levels, inflammation, and LDL oxidation, and with the burden of the most prevalent comorbidities in ESRD patients, diabetes and hypertension.

## 2. Material and Methods

### 2.1. Subjects

This study included patients from five dialysis clinics in the Northern Region of Portugal; the Committee on Ethics, from the Faculty of Pharmacy, Porto University, approved the study protocol. Patients and controls participated in the study after signing an informed and written consent, respecting their privacy rights. Patients with malignancy, autoimmune disease, and inflammatory or infectious diseases and with increased alanine transaminase and/or aspartate transaminase levels were excluded.

The study included 194 ESRD patients under chronic dialysis (28 on HD and 166 under online hemodiafiltration), for more than 90 days. Therapeutic dialysis was performed 3 times per week, for 3-5 hours, and patients were on dialysis treatment for a median period of 4.09 (1.74–7.60) years. Dialysis clearance of urea was expressed as *Kt*/*V*. The main causes of renal disease were diabetic nephropathy (*n* = 79), hypertensive nephrosclerosis (*n* = 32), chronic glomerulonephritis (*n* = 11), polycystic kidney disease (*n* = 8), other diseases (*n* = 26), and uncertain etiology (*n* = 38). Concerning vascular access, 26 patients used central venous catheter, 158 used an arteriovenous fistula, and 10 patients used an arteriovenous graft. Diabetes was established by current guidelines [18] and/or by the use of insulin or oral hypoglycemic drugs. Hypertension was defined by current guidelines [19] or by the use of hypertensive agents.

A group of 22 healthy volunteers was selected as control, based on normal hematological and biochemical values, and no history of kidney, inflammatory diseases or dyslipidemia.

### 2.2. Collection and Preparation of Blood Samples

Blood was collected immediately before dialysis procedure, by venepuncture, into tubes with and without anticoagulant (ethylenediaminetetraacetic acid), in order to obtain plasma and serum, respectively. Samples were processed within 2 hours of collection; aliquots of plasma and serum were prepared and immediately stored at -80°C until assayed.

### 2.3. Analytical Assays

Lipid profile was evaluated by routine procedures, using an autoanalyser (Cobas Integra 400 Plus, Roche Diagnostics, Basel, Switzerland) and commercially available kits; TC, TG, HDLc, and LDLc concentrations were determined by enzymatic colorimetric tests (Roche Diagnostics, Basel, Switzerland).

HDL and LDL subpopulations were separated and quantified using a Lipoprint® kit from Quantimetrix Corp. (Redondo Beach, CA, USA). This analysis involves a polyacrylamide gel electrophoresis followed by a complete data acquisition and quantification of lipoprotein subpopulations, using the Lipoprint System.

HDL is separated into 10 subfractions that are classified as large HDL particles (1-3 subfractions), intermediate HDL particles (4-7 subfractions), and small HDL particles (8-10 subfractions).

LDL particles are divided into 7 subpopulations, the LDL1 and LDL2 subpopulations corresponding to larger LDL particles and LDL3 to LDL7 subfractions corresponding to small LDL particles. Besides the LDL subfractions, the LDL Lipoprint profile also includes one band of VLDL, 3 midbands corresponding to intermediate-density lipoprotein (IDL), and one HDL band. The mean LDL particle size is determined.

Circulating levels of oxLDL, adiponectin, and leptin were evaluated by enzyme immunoassays (Oxidized LDL ELISA, Mercodia, Uppsala, Sweden, and human total adiponectin and human leptin, R&D Systems Inc., Minneapolis, USA, respectively); the oxLDL/LDLc ratio was calculated to assess the value of LDL oxidation within LDL particles. CRP values were measured by immunoturbidimetry (High-Sensitivity CRP (latex), Roche Diagnostics, Basel, Switzerland). PON1 activity was assessed spectrophotometrically and expressed in nmol of p-nitrophenol/ml/min. PON1 activity was evaluated by adding serum to 1 ml Tris/HCl buffer (100 mmol/l, pH 8.0) containing 2 mmol/l CaCl_2_ and 5.5 mmol/l paraoxon (O,O-diethyl-O-p-nitrophenylphosphate; Sigma Chemical Co.). The rate of generation of p-nitrophenol was determined at 412 nm, 37°C, via the use of a continuously recording spectrophotometer.

### 2.4. Statistical Analysis

Statistical analysis was performed using the Statistical Package for Social Sciences (SPSS, version 23.0, Chicago, IL, USA) for Windows. Kolmogorov-Smirnovanalysis was used to determine if data presented a normal distribution. Parameters presenting a non-Gaussian distribution are presented as median (interquartile range); for those with Gaussian distribution, results are presented as mean ± standard deviation. For comparisons between controls and patients, we used, for continuous variables, the Mann-Whitney *U* test and the unpaired Student *t*-test, in accordance with the Gaussian distribution of the variables; for categorical variables, chi-squared test and Fisher's exact test were employed. To compare the groups in terms of comorbidities, we used one-way ANOVA supplemented with Tukey post hoc test (for variables presenting a normal distribution). Adjustment for confounding factors (e.g., age, BMI, diabetes) was performed using analysis of covariance (ANCOVA; variable data respected a normal distribution). Data presenting a non-Gaussian distribution were transformed to data with normal distribution, by using the method described by Templeton [20]. Spearman's rank correlation coefficient was performed to evaluate relationships between sets of data. To evaluate the contribution of the different variables to adiponectin levels, a multiple regression analysis was performed, using stepwise selection (all variable data included in the regression analysis respected a normal distribution). A *P* value lower than 0.05 was considered as statistically significant.

## 3. Results

Controls and patients did not present significant differences in what concerns gender, weight, height, and BMI; clinical data for patients is showed in Table 1. Patients, as compared to controls, presented significantly lower values of TC, HDLc, LDLc, oxLDL, and LDLc/HDLc and significantly higher TG, CRP, and adiponectin levels (Table 1). Concerning HDL subfractions, patients presented higher percentage of large HDL and lower percentages of intermediate and small HDL. As compared to controls, ESRD patients also presented significantly higher VLDL and MID-B (%) and lower % of larger LDL (LDL 1 and LDL 2). Differences persisted significant after statistical adjustment for age.

**Figure 1:**
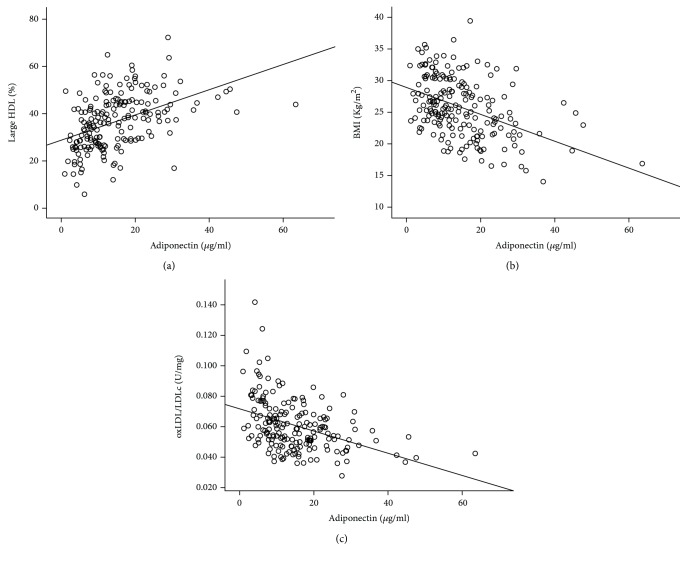
Correlations observed between adiponectin and (a) large high-density lipoprotein (HDL; *r* = 0.509, *P* < 0.001), (b) body mass index (BMI; *r* = −0.431, *P* < 0.001), and (c) oxidized low-density lipoprotein (oxLDL)/LDLc ratio (*r* = −0.434, *P* < 0.001) in end-stage renal disease patients on chronic dialysis.

Adiponectin correlated positively with large HDL (Figure 1(a)) and negatively with BMI (Figure 1(b)), oxLDL (*r* = −0.306, *P* < 0.001), and oxLDL/LDLc (Figure 1(c)), as well as with intermediate HDL (*r* = −0.374, *P* < 0.001), small HDL (*r* = −0.447, *P* < 0.001), VLDL (*r* = −0.388, *P* < 0.001), and leptin (*r* = −0.390, *P* < 0.001).

Leptin correlated inversely with large HDL (*r* = −0.286, *P* < 0.001) and positively with intermediate HDL (*r* = 0.212, *P* = 0.003), small HDL (*r* = 0.271, *P* < 0.001), VLDL (*r* = 0.209, *P* = 0.003), and BMI (*r* = 0.579, *P* < 0.001).

OxLDL and oxLDL/LDLc correlated significantly and negatively with large HDL (*r* = −0.401, *P* < 0.001; *r* = −0.240, *P* = 0.001, respectively) and positively with intermediate HDL (*r* = 0.253, *P* < 0.001; *r* = 0.143, *P* = 0.007, respectively), small HDL (*r* = 0.390, *P* < 0.001; *r* = 0.232, *P* = 0.001, respectively), VLDL (*r* = 0.281, *P* < 0.001; *r* = 0.196, *P* = 0.006, respectively), and BMI (*r* = 0.186, *P* = 0.009; *r* = 0.252, *P* < 0.001, respectively). OxLDL also correlated with LDL size (*r* = −0.251, *P* < 0.001), CRP (*r* = 0.143, *P* = 0.047), and leptin (*r* = 0.271, *P* < 0.001).

BMI correlated with large HDL (*r* = −0.379, *P* < 0.001), intermediate HDL (*r* = 0.276, *P* < 0.001), small HDL (*r* = 0.346, *P* < 0.001), and PON1/HDLc (*r* = 0.295, *P* < 0.001).

In multiple linear regression, after transformation to variables with normal distribution, large HDL (*β* = 0.322; *P* < 0.001) and oxLDL/LDLc (*β* = −0.288; *P* = 0.001), as well as BMI (*β* = −0.308; *P* < 0.001), remained significantly associated with adiponectin.

**Figure 2:**
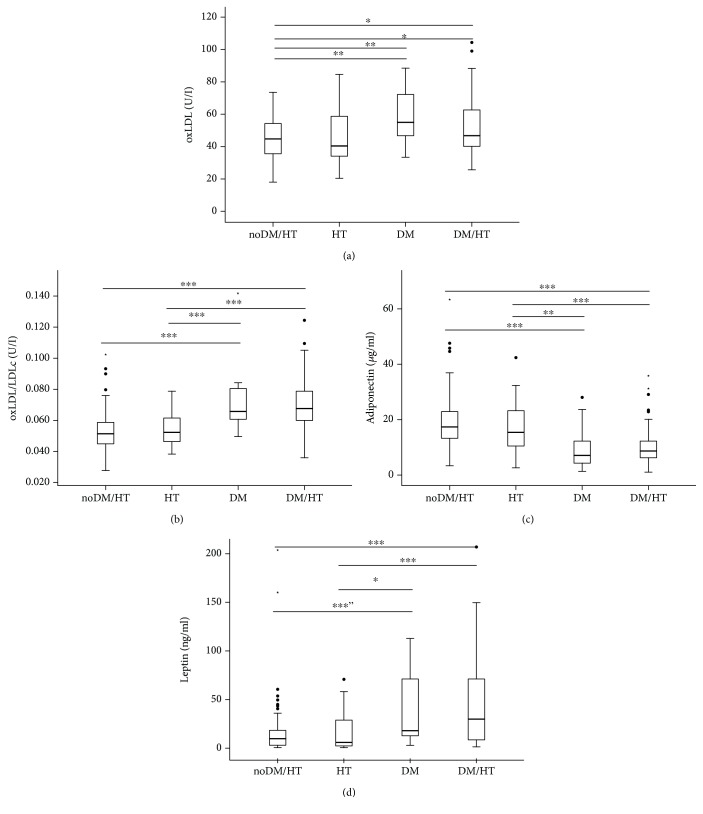
Levels of oxidized low-density lipoprotein (oxLDL) (a), oxLDL/LDLc (b), adiponectin (c), and leptin (d), for end-stage renal disease (ESRD) patients on chronic dialysis, according to the more prevalent comorbidities, diabetes mellitus (DM) and hypertension (HT). ^∗^*P* < 0.05; ^∗∗^*P* < 0.01; ^∗∗∗^*P* < 0.001; ^″^loss of significance after statistical adjustment for body mass index.

Concerning the most frequent comorbidities in ESRD patients, diabetes and hypertension, we analyzed our data according to the association with those comorbidities. We found that 17 patients presented only DM, 38 presented only HT, 70 presented simultaneously DM and HT (DM+HT), and 69 patients did not present DM nor HT. Concerning the use of statins, 58.8% in the DM group, 44.7% in the HT group, 68.6% in the DM+HT group, and 43.5% of the patients that did not present DM or HT were on statin therapy. The four groups did not differ significantly in age and gender but differed in BMI, which was higher in DM and DM+HT groups (Table 2). Patients with DM and with DM+HT, as compared to patients without DM and HT or patients with only HT, presented higher values of oxLDL, oxLDL/LDLc, and leptin and lower adiponectin (Figure 2). Patients with DM, as compared to patients without DM and HT, after statistical adjustment for BMI, presented higher intermediate HDL; the significance for leptin was lost. Patients with DM+HT, as compared to the group without DM or HT, presented, after statistical adjustment for BMI, higher oxLDL/LDLc, adiponectin, leptin, and MID-A percentage and lower TC, HDLc, LDLc, and CRP. Patients with HT, as compared to those with only DM, revealed higher HDLc and large HDL and lower TG, PON1/HDLc, and intermediate HDL. Patients with DM+HT, as compared to the group of patients with only HT, presented, after statistical adjustment for BMI, higher values of TG, oxLDL, oxLDL/LDLc, and leptin and lower CRP and adiponectin (Table 2). There were no statistically significant differences for the studied variables between patients with HT and patients without DM or HT.

We also analyzed our data according to BMI categories (Table 3). The groups did not differ significantly in what concerns age, gender, and number of cases of HT but differed in the number of cases of diabetes; the underweight (BMI < 18.5 kg/m^2^) patients were not diabetic; the normal weight (BMI 18.5-24.9 kg/m^2^) group had lower number of cases of diabetes (33%) than the overweight (BMI 25-29.9 kg/m^2^) and obese (BMI ≥ 30 kg/m^2^) groups (43% and 58%, respectively). Regarding statin therapy, 50.0% of underweight patients, 40.7% of normoponderal patients, 66.7% of overweight patients, and 62.2% of obese patients were on statin therapy.

In the underweight group, the number of patients was small (*n* = 8), compromising statistical evaluation; therefore, we did not analyze data from this group.

Overweight (*n* = 60) and obese (*n* = 45) patients, as compared to normoponderal patients (*n* = 81), showed significantly lower HDLc, adiponectin, and % of large HDL and significantly higher TG, oxLDL/LDLc, PON1/HDLc, leptin, % of VLDL, and small HDL. Obese patients also presented significantly higher % of intermediate HDL, oxLDL, TC/HDLc, and LDLc/HDLc than normal weight patients. After statistical adjustment for diabetes, the statistical differences observed for the overweight group were lost; in the obese group, differences found for HDLc, PON1/HDLc, adiponectin, leptin, VLDL, large HDL, intermediate HDL, and small HDL persisted. Obese, as compared to overweight patients, presented higher values of leptin and % of small HDL and lower large HDL; after statistical adjustment for diabetes, only the differences for leptin remained significant (Table 3).

## 4. Discussion

In this work, we studied in ESRD patients the modifications in LDL and HDL profiles and their relationship with BMI, adipokine levels, inflammation, and LDL oxidation and with the coexistence of DM and HT, the most prevalent comorbidities in these patients.

We found that ESRD patients on HD presented some significant risk changes in lipid profile, namely, decreased HDLc and increased TG values, alongside with protective changes, such as decreased TC and LDLc, as compared to the control group.

An increase in small HDL subpopulations was found in patients with coronary artery disease (CAD) [9]. Patients with acute coronary syndrome (ACS) and with stable CAD showed dysfunctional HDL subfractions that have distinct composition and diminished anti-inflammatory potential, especially for HDL3 in ACS, which can be used to discriminate between these two conditions [21]. In a Chinese cohort, it was found that large HDL subpopulation correlated negatively to very early CAD [22]. Higher values of small LDL particles and lower values of large HDL particles, as well as a reduction in LDL size, were reported in incident diabetes [10]. Decreased large HDL particles and increased small HDL particles were found in patients with hypertension [23]. However, as previously mentioned, it is still a matter of debate which subfraction of HDL is more atheroprotective [6], with reports referring a positive association between HDL3 and protection from CAD [24], in opposition to the above-referred reports in the opposite direction. In spite of the lower circulating levels of HDL, the study of its subfractions showed a less atherogenic profile in patients than in controls, presenting significantly higher percentage of large HDL particles and lower intermediate and small HDL subfractions. These results are in accordance with published data [11–13]. Nevertheless, it has been reported that the content of HDL in triglycerides and phospholipids appears to be altered in CKD patients, leading to impairment in cholesterol efflux [25]. Gluba-Brzozka et al. [12] found the same changes in HDL subfractions and also proposed that large HDL may have a unique proteome and lipid composition, associated with an impaired cholesterol efflux capacity. This lack of HDL functionality may contribute to the paradoxical coexistence of increased large HDL and enhanced risk for CVD-related events in ESRD patients.

In accordance with others [15, 26], we also found in HD patients significantly higher levels of adiponectin (more than twofold the control value), known for its beneficial cardiovascular, antiatherogenic, anti-inflammatory, and antidiabetic properties. In obesity, type 2 DM, and coronary artery disease, adiponectin levels appear to be reduced [27].

The adiponectin circulating levels are increased in HD patients; however, it is still unclear if its effects on metabolism remain intact. It has been hypothesized that the uremic milieu, especially high in HD patients, may induce the development of adiponectin resistance [28]. Another raised explanation is that the enhanced adiponectin levels are due to a decreased renal excretion, in ESRD [29]. It was also reported that adiponectin may be secreted to alleviate inflammatory or vascular injuries, although this counterregulation may not be efficient enough, due to the effects of the proatherogenic uremic environment [15].

We found that adiponectin correlated positively and significantly with large HDL and negatively with intermediate and small HDL. Our data suggest that the enhancement in adiponectin induces a more protective HDL profile, as showed by the increase in large HDL and the decrease in intermediate and small HDL particles. However, since HDLc levels are low, these differences in HDL quality (size and composition) may not produce a significant impact in CVD risk, in HD patients. Besides, we cannot rule out the possibility that the alterations in size and composition are not accompanied by improvement in HDL functionality.

Adiponectin is known to correlate inversely with adiposity, even in ESRD patients [16]. It was also reported that in HD patients, adiponectin may have a role in improving oxidative stress that is common in these patients [30]. In agreement, we observed a significant inverse correlation of adiponectin with oxLDL, oxLDL/LDLc, and BMI, in dialysis patients. By performing a multiple linear regression analysis, we found that the best predictors of adiponectin levels were, besides BMI, large HDL and oxLDL/LDLc. It appears that in ESRD patients on dialysis, adiposity and adiponectin, large HDL, and LDL oxidation within LDL particles are tightly interrelated.

Concerning antioxidant properties of HDL, we found that large HDL was significantly and inversely correlated with oxLDL and oxLDL/LDLc, while intermediate and small HDL subfractions were positively correlated with oxidative changes in LDL, suggesting that HDL subfractions may have different roles in what concerns antioxidant activity.

In the evaluation of LDL fractions and subfractions, we found in dialysis patients, as compared to controls, significantly lower (%) larger LDL (LDL 1 and 2) and similar (%) small LDL particles. Our data are in accordance with other reports [11–13] showing the same changes in HD patients and proposing that small dense LDL particles are not associated with CVD in these patients [31].

CKD has been associated with hypertriglyceridemia, impairment of VLDL catabolism, due to lipoprotein lipase deficiency/dysfunction [32], and IDL has been reported as an independent risk factor for atherosclerosis [33]. In accordance, we observed higher values of TG, VLDL, and IDL-B in ESRD patients. The lower HDLc levels in ESRD, also observed in our patients, have been associated with a decrease in apolipoprotein A-1 production and in lecithin-cholesterol acyltransferase activity [34].

Diabetes and hypertension are common comorbidities in HD patients and may contribute to the high CVD risk in these patients. In that way, we analyzed our patients according to the coexistence of such comorbidities.

We found that patients with DM presented a different and less protective lipid profile than the other patients under chronic dialysis. Patients with DM and DM+HT, as compared to dialysis patients without DM, presented several significant risk changes, namely, higher BMI scores, as well as higher values of TG, oxLDL, oxLDL/LDLc, and leptin, and lower HDLc, large HDL subfraction, and adiponectin. The HD patients presenting only HT and the patients without DM or HT showed a similar and more protective profile. These findings suggest that these comorbidities (DM and HT) contribute differently to CV risk profile. In accordance with our data, Likely et al. [16] also reported lower adiponectin values in diabetic ESRD patients, when compared with nondiabetic patients, although higher than in controls. Considering that DM and DM+HT patients presented a significantly higher BMI, as compared to the other two groups, we performed a statistical adjustment for BMI; the differences in oxLDL, oxLDL/LDLc, and adiponectin between diabetic and nondiabetic ESRD patients persisted significantly (Figure 2). It is known that oxidative modifications in LDL play a crucial role in the initiation and progression of atherosclerosis [35] and are accepted as a marker for CVD risk. The significant enhancement in LDL oxidation in the diabetic patients, as well as the reduced values of adiponectin, may contribute to the poor outcome [36] and health-related quality of life [37] reported for diabetic patients with ESRD.

In considering the importance of obesity as a predisposing factor for diabetes and for CVD, we analyzed our data according to BMI categories and, afterwards, performed a statistical adjustment for diabetes. The obese patients presented the less protective CVD risk profile, with significantly lower HDLc, % of large HDL, and adiponectin and significantly higher (%) small HDLc subfractions and leptin. These changes persisted significantly after adjustment for diabetes, suggesting that in HD patients, the values of HDLc and HDL subfractions are tightly related with adiposity.

High levels of leptin, associated with reduction in its activity, are known to lead to severe insulin resistance [38]. Hyperleptinemia is also known to correlate with progression of CKD [17]. Hypoadiponectinemia induces a decrease in glucose uptake in the skeletal muscle, an increase in hepatic gluconeogenesis, and a diminishment in fatty acid oxidation favoring insulin resistance and type 2 DM [39]. Higher adiposity is known to associate with a decrease in adiponectin and an increase in leptin levels [40, 41]. The differences we found in adipokines levels suggest that in ESRD patients under chronic dialysis, diabetes and higher BMI associate with lower adiponectin and with an enhancement in leptin levels. In spite of the higher CRP values in ESRD patients, no correlations were found with the coexistence of comorbidities and with BMI categories, suggesting that inflammation is particularly linked to renal disease and/or dialysis.

Statins, known to lower cholesterol and LDLc levels, possess other effects, such as endothelial function improvement and antioxidant and anti-inflammatory properties. According to the literature, the effects of statin on HDL subfractions are especially related with subfraction composition [42–44]; concerning the effect of statins on the size of LDL subfractions, data is controversial [42, 45]. Nonetheless, in our study, the number of diabetic, overweight, and obese patients on statin therapy was higher than in the other groups analyzed. Thus, the use of statins appears to have an irrelevant impact in the changes observed on the HDL subfractions and adipokine profile.

In summary, in ESRD patients, the higher adiponectin levels seem to favor HDL modifications, from smaller to larger subfractions, and antioxidant protection for LDL particles. ESRD diabetic patients presented higher values of oxLDL, oxLDL/LDLc, and leptin and lower adiponectin than nondiabetic patients; obese patients revealed lower HDLc and adiponectin, higher leptin and a more atherogenic HDL subfractions profile.

Our data suggest that the coexistence of DM and adiposity in ESRD patients on dialysis contributes to a higher CVD risk, as showed by their lipid and adipokine profiles.

## Figures and Tables

**Table 1:** Demographic and clinical data, lipid profile, oxidized low-density lipoprotein (oxLDL), paraoxonase (PON) 1 activity, C-reactive protein (CRP), adipokines, and fractions and subfractions of lipoproteins, for the control group and end-stage renal disease (ESRD) patients under chronic dialysis.

	Controls (*n* = 22)	ESRD patients (*n* = 194)	*P* value
*Demographic and clinical data*			
Age (years)	56.9 (52.3–59.8)	71.3 (60.0–79.6)	<0.001
Gender	14 F/8 M	87 F/107 M	0.095
Weight (kg)	65.5 (58.8–80.3)	68.7 (58.5–78.7)	0.983
Height (cm)	170 (157–175)	162 (157–170)	0.078
BMI (kg/m^2^)	24.3 ± 3.4	25.8 ± 4.7	0.073
Systolic blood pressure (mmHg)	123 ± 10	137 ± 22	<0.001
Diastolic blood pressure (mmHg)	80 ± 7	62 ± 13	<0.001
Dialysis length (years)	—	4.09 (1.74–7.60)	—
URR (%)	—	79.0 (75.8–83.0)	—
*Kt*/*V*	—	1.82 ± 0.31	—
Ultrafiltration volume (ml)	—	2371 ± 919	—
*Biochemical data*			
TC (mg/dl)	202 ± 23	160 ± 39	<0.001
HDLc (mg/dl)	52.6 (44.7–65.4)	46.0 (39.2–56.6)	0.022
LDLc (mg/dl)	122 (104–138)	79 (63–103)	<0.001
TG (mg/dl)	90.0 (71.3–107.5)	130.5 (97.5–178.5)	<0.001
TC/HDLc	3.70 (3.03–4.66)	3.31 (2.71–4.10)	0.119
LDLc/HDLc	2.27 (1.58–2.87)	1.73 (1.28–2.30)	0.014
oxLDL (U/l)	67.5 (55.5–73.5)	46.5 (36.5–60.0)	<0.001
oxLDL/LDLc (U/mg)	0.055 (0.049–0.064)	0.059 (0.050–0.069)	0.310
PON1 (nmol of p-nitrophenol/ml/min)	413 (386–467)	405 (361–473)	0.579
PON1/HDLc	8.43 (6.23–9.94)	9.05 (6.99–11.15)	0.107
CRP (mg/dl)	0.10 (0.04–0.21)	0.37 (0.15–0.72)	<0.001
Adiponectin (*μ*g/ml)	5.75 (3.02–8.35)	12.28 (7.86–19.25)	<0.001
Leptin (ng/ml)	9.3 (4.9–20.6)	14.0 (5.1–41.5)	0.144
*Lipoprotein fractions/subfractions*			
VLDL (%)	14.3 ± 3.0	18.7 ± 4.0	<0.001
MID-C (%)	15.1 ± 2.4	16.1 ± 2.3	0.054
MID-B (%)	6.6 (5.9–7.7)	7.8 (6.6–9.0)	0.004
MID-A (%)	6.3 (5.6–8.5)	6.8 (5.4–8.4)	0.941
LDL 1 (%)	18.3 (16.3–20.0)	13.0 (10.7–15.4)	<0.001
LDL 2 (%)	9.9 (7.4–13.0)	8.1 (5.6–10.9)	0.036
LDL 3-7 (%)	0.7 (0.0–1.1)	0.7 (0.0–1.7)	0.789
HDL (%)	27.0 (24.0–31.2)	26.8 (23.2–30.2)	0.793
LDL size	271 (270–272)	270 (268–272)	0.265
*HDL subfractions*			
Large HDL (%)	24.7 (22.3–31.6)	37.2 (28.3–44.9)	<0.001
Intermediate HDL (%)	48.8 (45.9–50.6)	45.6 (41.2–49.8)	0.013
Small HDL (%)	24.9 (17.5–29.5)	17.4 (12.6–22.7)	0.001

F: female; M: male; BMI: body mass index; URR: urea reduction ratio; TC: total cholesterol; HDLc: high-density lipoprotein cholesterol; TG: triglycerides; VLDL: very-low-density lipoprotein; MID: midbands (these comprise intermediate-density lipoprotein (IDL)); LDL 3–7 corresponds to small LDL subfractions. Values are presented as median (interquartile range) or as mean ± standard deviation (SD).

**Table 2:** Body mass index (BMI), lipid profile, paraoxonase (PON) 1 activity, C-reactive protein (CRP), and fractions and subfractions of lipoproteins, for end-stage renal disease (ESRD) patients on chronic dialysis, according to the more prevalent comorbidities, diabetes mellitus (DM) and hypertension (HT).

	noDM/HT (*n* = 69)	HT (*n* = 38)	DM (*n* = 17)	DM+HT (*n* = 70)
BMI (kg/m^2^)	24.1 ± 4.3	25.0 ± 5.4	28.0^aa^ ± 4.7	27.4^aaa^ ± 4.0^b^
*Biochemical data*				
TC (mg/dl)	170 ± 39	164 ± 37	156 ± 33	149^aa^ ± 38^b,^^∗^
HDLc (mg/dl)	50.6 (43.8–59.6)	46.1 (40.0–61.9)	39.7^aa,^^∗^ (34.8–47.8)^b^	43.0^aa^^∗^ (36.9–53.8)^∗^
LDLc (mg/dl)	88 (68–107)	77 (65–112)	82 (64–105)	^a^72 (57–91)
TG (mg/dl)	121.0 (96–165.5)	115.0 (85.0–141.5)	189.0^a,^^∗^ (116.0–258.0)^bb^	141.0^a,^^∗^ (98.9–195.0)^b^
TC/HDLc	3.37 (2.71–4.04)	3.40 (2.44–4.23)	3.38 (3.00–4.98)	3.15 (2.74–4.04)
LDLc/HDLc	1.80 (1.28–2.27)	1.83 (1.06–2.41)	1.79 (1.65–3.10)	1.67 (1.29–2.16)
PON1 (nmol of p-nitrophenol/ml/min)	410 (368–476)	384 (350–472)	431 (355–480)	398 (362–466)
PON1/HDLc	8.74 (6.52–10.13)	8.45 (6.32–10.92)	10.38^a,^^∗^ (9.00–13.86)^b^	9.73^a,^^∗^ (7.41–11.74)
CRP (mg/dl)	0.43 (0.18–0.74)	0.46 (0.19–0.99)	0.53 (0.24–0.93)	0.24^a^ (0.11–0.48)^b^
*Lipoprotein fractions/subfractions*				
VLDL (%)	18.1 ± 3.7	17.8 ± 4.0	20.1 ± 4.2	19.4 ± 4.1
MID-C (%)	16.0 ± 2.0	16.0 ± 2.5	16.7 ± 2.1	16.2 ± 2.4
MID-B (%)	7.8 (6.8–8.7)	7.2 (6.1–8.3)	7.3 (6.4–9.4)	8.3 (6.8 -10.1)^b^
MID-A (%)	6.5 (5.1–7.6)	6.8 (5.9–8.2)	5.7 (4.6–8.8)	7.4 (5.6–8.9)^a^
LDL 1 (%)	12.8 (10.7–15.0)	13.5 (10.6–15.9)	12.4 (9.8–14.0)	13.3 (11.3–15.9)
LDL 2 (%)	8.3 (6.2–11.6)	8.2 (4.4–10.5)	7.8 (6.2–11.2)	8.0 (5.1–9.9)
LDL 3-7 (%)	0.7 (0.0–1.6)	0.4 (0.0–1.7)	0.9 (0.0–4.6)	0.8 (0.0–1.6)
HDL (%)	27.6 (24.3–30.8)	28.4 (22.6–33.8)	25.3^a^ (22.2–27.0)	24.8^aa^ (21.9–29.4)^b,^^∗^
LDL size	270 (268–272)	270 (268–273)	271 (264–272)	270 (268–272)
*HDL subfractions*				
Large HDL (%)	40.6 (29.4–46.2)	39.6 (29.3–48.7)	35.0^a,^^∗^ (21.1–39.6)^b^	33.2^aa,^^∗^ (26.0–41.6)^b,^^∗^
Intermediate HDL (%)	45.0 (41.2-48.6)	43.7 (39.4–48.9)	50.0^a^ (41.9–51.0)^b^	46.4 (43.1–50.4)
Small HDL (%)	15.0 (11.1–21.6)	16.4 (12.0–21.3)	21.9 (12.8–21.7)	19.1^a,^^∗^ (13.9–24.0)

TC: total cholesterol; HDLc: high-density lipoprotein cholesterol; LDLc: low-density lipoprotein cholesterol; TG: triglycerides; VLDL: very-low-density lipoprotein; MID: midbands (these comprise intermediate-density lipoprotein (IDL)); LDL 3–7 corresponds to small LDL subfractions. Values are presented as median (interquartile range) or as mean ± standard deviation (SD); ^a^*P* < 0.05, ^aa^*P* < 0.01, and ^aaa^*P* < 0.001 vs. ESRD patients without DM or HT (noDM/HT); ^b^*P* < 0.05, ^bb^*P* < 0.01, and ^bbb^*P* < 0.001 vs. ESRD patients with HT; ^∗^loss of significance after statistical adjustment for BMI.

**Table 3:** Lipid profile, oxidized low-density lipoprotein (oxLDL), paraoxonase (PON) 1 activity, C-reactive protein (CRP), adipokines, and fractions and subfractions of lipoproteins for end-stage renal disease (ESRD) patients on chronic dialysis, according to body mas index (BMI) groups.

	BMI < 18.5 kg/m^2^(*n* = 8)	BMI 18.5–24.9 kg/m^2^ (*n* = 81)	BMI 25–29.9 kg/m^2^ (*n* = 60)	BMI ≥ 30 kg/m^2^ (*n* = 45)
*Biochemical data*				
TC (mg/dl)	171 ± 28	162 ± 40	157 ± 40	159 ± 36
HDLc (mg/dl)	54.4 (53.1–83.1)	49.4^a,^^∗^ (41.8–59.8)	44.5^aa^ (36.3–54.1)^b,^^∗^	40.9^aaa^ (34.8–48.7)^bbb^
LDLc (mg/dl)	62 (79–94)	77 (64–108)	79 (59–101)	80 (63–103)
TG (mg/dl)	111.5 (74.8–140.0)	116.0 (83.5–161.0)	134.5 (99.0–194.0)^b,^^∗^	156.0^a,^^∗^ (114.0–237.0)^bb,^^∗^
TC/HDLc	2.96 (2.17–3.17)	3.13 (2.52–3.74)	3.24 (2.76–4.21)	3.96^a^^∗^ (3.03-4.26)^bb,^^∗^
LDLc/HDLc	1.39 (0.87–1.68)	1.71 (1.18–2.10)	1.71 (1.30–2.40)	2.01^a,^^∗^ (1.44–2.42)^b,^^∗^
oxLDL (U/l)	45.8 (29.2–52.2)	44.6 (36.1–55.8)	49.6 (35.3–62.9)	50.0 (41.5–63.7)^b,^^∗^
oxLDL/LDLc (U/mg)	0.052 (0.045–0.065)	0.047 (0.054–0.066)	0.052 (0.060–0.075)^b,^^∗^	0.064 (0.051–0.076)^b,^^∗^
PON1 (nmol of p-nitrophenol/ml/min)	379 (339–464)	393 (362–459)	423 (353–488)	399 (368–465)
PON1/HDLc	6.56 (4.11–8.79)	8.31 (6.38–10.35)	9.54^a,^^∗^ (7.69–11.20)^b,^^∗^	9.99^aa,^^∗^ (8.68–12.13)^bb^
CRP (mg/dl)	0.44 (0.21–2.08)	0.32 (0.12–0.71)	0.36 (0.18–0.74)	0.42 (0.19–0.75)
Adiponectin (ug/ml)	28.65 (20.93–35.69)	16.24^aa^ (9.73–23.56)	11.48^aaa^ (7.25–16.20)^bb,^^∗^	8.23^aaa^ (5.43–12.78)^bbb^
Leptin (ng/ml)	3.40 (0.70–7.37)	6.50 (2.44–17.36)	17.42^aa,^^∗^ (6.96–49.19)^bbb,^^∗^	43.08^aaa^ (20.31–97.09)^bbb,ccc^
*Lipoprotein fractions/subfractions*				
VLDL (%)	16.4 ± 2.2	17.6 ± 3.7	19.2^a,∗^ ± 4.1^b,∗^	20.3^aa,∗^ ± 4.1^bbb^
MID-C (%)	15.2 ± 2.3	15.8 ± 2.3	16.6 ± 2.3	16.2 ± 2.0
MID-B (%)	6.9 (5.6–7.5)	7.8 (6.3–8.8)	7.9^a,^^∗^ (7.0–9.6)	7.7^a,^^∗^ (7.0-9.5)
MID-A (%)	6.2 (4.1–7.5)	6.6 (5.3–8.4)	6.9 (5.6–8.8)	6.9 (5.2–8.3)
LDL 1 (%)	12.3 (9.4–13.9)	13.4 (11.2–16.2)	13.0 (10.3–15.7)	12.8 (10.9–15.2)
LDL 2 (%)	7.2 (6.4–11.0)	8.2 (5.4–11.1)	7.8 (5.4–10.0)	8.4 (5.8–11.7)
LDL 3-7 (%)	1.2 (0.9–1.6)	0.0 (0.0–1.8)	0.7 (0.0–1.8)	0.8 (0.0–2.0)
HDL (%)	31.1 (29.6–38.2)	27.4 (24.2–32.3)	26.7^a^ (22.7–29.9)^bb^	25.0^aa^ (21.8–27.6)
LDL size	270 (269–270)	271 (268–272)	270 (268–273)	270 (267–272)
*HDL subfractions*				
Large HDL (%)	43.8 (40.5–47.7)	40.7 (30.0–49.3)	36.6^a,^^∗^ (26.6–43.2)^b,^^∗^	28.6^a,^^∗^ (24.7–37.9)^bbb; c,^^∗^
Intermediate HDL (%)	43.3 (39.8-45.3)	44.3 (39.8–48.2)	46.3 (40.7–50.0)	48.5^a,^^∗^ (44.5–50.5)^bb,^
Small HDL (%)	13.3 (11.0–16.7)	14.1 (10.6–21.0)	17.4 (13.5–22.4)^b,^^∗^	21.2^a,^^∗^ (15.9–27.3)^bbb; c,^^∗^

TC: total cholesterol; HDLc: high-density lipoprotein cholesterol; TG: triglycerides; VLDL: very-low-density lipoprotein; MID: midbands (these comprise intermediate-density lipoprotein (IDL)); LDL 3–7 corresponds to small LDL subfractions. Values are presented as median (interquartile range) or as mean ± standard deviation (SD); ^a^*P* < 0.05, ^aa^*P* < 0.01, and ^aaa^*P* < 0.001 vs. ESRD patients with BMI < 18.5 kg/m^2^; ^b^*P* < 0.05, ^bb^*P* < 0.01, and ^bbb^*P* < 0.001 vs. ESRD patients with BMI 18.5–24.9 kg/m^2^; ^c^*P* < 0.05, ^cc^*P* < 0.01, and ^ccc^*P* < 0.001 vs. ESRD patients with BMI 24.9–29.9 kg/m^2^; ^∗^loss of significance after statistical adjustment for diabetes.

## Data Availability

The data used to support the findings of this study are included within the article.
